# Clinical study on the relationship between hepatitis B virus infection and risk of breast cancer: a large sized case-control and single center study in southwest of China

**DOI:** 10.18632/oncotarget.19132

**Published:** 2017-07-10

**Authors:** Lin-Jie Lu, Vishnu Prasad Adhikari, Chun-Xia Zhao, He Wu, Wei Dai, Xin Li, Hong-Yuan Li, Guo-Sheng Ren, Kai-Nan Wu, Ling-Quan Kong

**Affiliations:** ^1^ Department of Endocrine and Breast Surgery, The First Affiliated Hospital of Chongqing Medical University, Chongqing 400016, China; ^2^ Department of Thyroid and Breast Surgery, Liuzhou People's Hospital, Liuzhou 545006, China

**Keywords:** breast cancer, hepatitis B virus, risk factor, etiology, breast oncohepatology

## Abstract

**Purpose:**

Chronic hepatitis C virus (HCV) infection is reported to be associated with early-onset breast cancer, while, as a hepadnavirus, hepatitis B virus(HBV) infection is more common than HCV in China. In this article, it is aimed to study the relationship between HBV infection and risk of breast cancer in China.

**Methods:**

The clinical data of 2452 cases of initially diagnosed breast cancer and 1926 cases of benign breast disease (as controls) with the consecutive reports of HBV serological markers and liver function tests, available in the Electronic Medical Records of the Breast Cancer Center of Chongqing, the southwest of China, from January 2011 to March 2015, were collected for analysis.

**Results:**

The average age of the initially diagnosed breast cancer patients was 50.3±11.3 years with the age peaking about 40- 49yeaers (39.7%). The positive rate (8.2%) of hepatitis B surface antigen in breast cancer patients was relatively higher than that (7.8%) in controls (*P*>0.05). While, the positive rate (66.4%)of hepatitis B core antibody in breast cancer patients was significantly higher than that (53.7%) in controls (*P*<0.05), so were the similar results in the age groups of 40-49 years, after multiple layer analysis stratified by age and compare HBV markers adjusting age with binary logistic regression. Meanwhile, the status of albumin, aminotransferase and aspartate transaminase (41.4 g/L, 22.9 U/L, 22.0 U/L) in breast cancer patients were significantly poorer than those (44.1 g/L,16.8 U/L, 19.2 U/L) in controls (*P*<0.05).

**Conclusions:**

Exposure to HBV infection may be a risk factor for breast cancer and may be also related to the earlier age onset of breast cancer (peaked around 40-49 years) among Chinese females.

## INTRODUCTION

Breast cancer is the most commonly diagnosed cancer and also a leading cause of cancer death among females worldwide. Breast cancer alone accounts for 25% of all cancer cases and 15% of all cancer deaths among females [1]. Though the incidence of breast cancer in China is lower than that of Western countries, the mean age of breast cancer patients in China was around the mid-40s, which was about a decade earlier than what was reported for western Caucasian women [2]. Furthermore, in China, female breast cancer peaked in age group of 40-49 years, while breast cancer clusters peaked around 60-69 years in western countries [2]. This will severely threatens the health and the quality of life among Chinese females. So more etiological researches on breast cancer, especially on the Chinese populace, should be carried out. The Nobel Laureate, Zur Hausen, H., considered that slightly more than 20% of global cancer burden can currently be linked to infectious agents, including viruses, bacteria, and parasites, meanwhile breast cancer remains an interesting candidate for a viral etiology [3]. Some viruses are suspected to play a role in the initiation or promotion of breast cancer [4–6]. A population-based case-control study about association between chronic viral hepatitis infection and breast cancer risk suggested that chronic hepatitis C virus (HCV) infection was associated with early-onset breast cancer [7]. Similarly, as a hepadnavirus, hepatitis B virus( HBV) infection is more common than HCV in China, where every surgical patient should undergo routine examination of HBV serological markers and liver function tests for peri-operative preparation before operation. Though with the nationwide vaccination program since 1992, the epidemiology of HBV infection in China is still moderate endemic now [8]. While there are few studies done on the relationship between HBV infection and the risk of breast cancer in China. In this paper, the status of HBV infection and liver function among initially diagnosed breast cancer patients as well as benign breast diseases patients (as controls) who had undergone surgical operation with pathological diagnosis was studied to explore whether the HBV infection is related with the risk and earlier age onset of breast cancer among Chinese females.

## RESULTS

**Figure 1:**
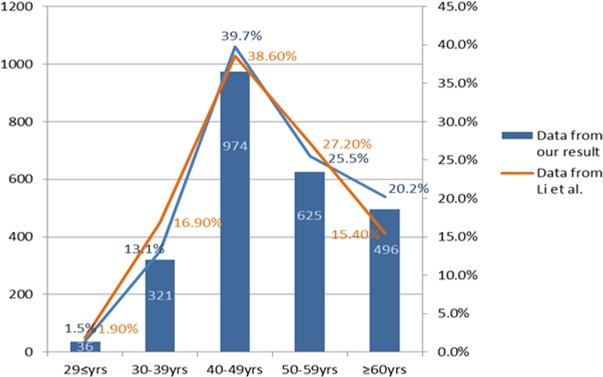
The age distribution of breast cancer patients at initial diagnosis with comparision of the data from Li et al [2]

There were 2452 cases of initially diagnosed breast cancer and 1926 cases of as controls with the consecutive reports of HBV serological markers enrolled in this study. The average age of the initially diagnosed breast cancer patients was 50.5±11.3 years, with age peaks being 40-49(39.7%)years old. (Table 1 and Figure 1)

**Table 1:** The age distribution of breast cancer patients and benign breast disease patients at initial diagnosis

Groups	Benign breast disease patients(n=1926)		Breast cancer patients(n=2452)	
Mean ± SD	36.7±11.2		50.5±11.3*	
Range	11-79		21-92	
29≤	564	(29.3%)	36	(1.5%)
30-39	559	(29.0%)	321	(13.1%)
40-49	607	(31.5%)	974	(39.7%)
50-59	145	(7.5%)	625	(25.5%)
≥60	51	(2.6%)	496	(20.2%)

* *p*< 0.05

Among 2452 cases of breast cancer, HBsAg was positive in 201 patients (8.2%), HBeAg positive in 18 patients (0.7%), HBcAb positive in 1629 patients (66.4%), HBsAb positive in 1403 patients (57.2%), HBeAb positive in 793 (32.3%); Among 1926 cases of controls, HBsAg was positive in 150 cases (7.8%), HBeAg positive in 21 cases (1.1%), HBcAb positive in 1035 patients (53.7%), HBsAb positive in 1156 patients (60%), HBeAb positive in 530 patients (27.5%), the positive rates of HBcAb and HBeAb were significantly higher than those in control group (*p*<0.05) (Table 2).

**Table 2:** The distribution patterns of HBV infection in breast cancer patients and benign breast disease patients

Serological markers of HBV					Breast cancer patients(n=2452)		Benign breast diseasepatients (n=1926)	
HBsAg	HBsAb	HBeAg	HBeAb	HBcAb	n	%	n	%
+	+/−	+/−	+/−	+/−	201	(8.2%)	150	(7.8%)
+/−	+	+/−	+/−	+/−	1403	(57.2%)	1156	(60.0%)
+/−	+/−	+	+/−	+/−	18	(0.7%)	21	(1.1%)
+/−	+/−	+/−	+	+/−	793	(32.3%)*	530	(27.5%)
+/−	+/−	+/−	+/−	+	1629	(66.4%)*	1035	(53.7%)
-	-	-	-	-	519	(21.2%)	477	(24.8%)
+	-	-	-	-	0	(0%)	1	(0.1%)
-	+	-	-	-	296	(12.1%)	398	(20.7%)
-	+	+	-	-	0	(0%)	1	(0.1%)
-	-	-	+	-	0	(0%)	2	(0.1%)
+	-	-	+	-	0	(0%)	1	(0.1%)
-	+	-	+	-	8	(0.3%)	11	(0.6%)
-	-	-	-	+	224	(9.1%)	98	(5.1%)
+	-	-	-	+	6	(0.2%)	7	(0.4%)
-	+	-	-	+	599	(24.4%)	394	(20.5%)
+	+	-	-	+	0	(0%)	1	(0.1%)
+	-	+	-	+	14	(0.6%)	18	(0.9%)
+	+	+	-	+	1	(0.04%)	1	(0.1%)
-	-	-	+	+	108	(4.4%)	50	(2.6%)
+	-	-	+	+	175	(7.1%)	115	(6%)
-	+	-	+	+	497	(20.3%)	345	(17.9%)
+	+	-	+	+	2	(0.1%)	5	(0.3%)
+	-	+	+	+	3	(0.1%)	1	(0.1%)

* *p*< 0.05

Multiple layer analysis stratified by age and compare HBV markers after adjusted for age with binary logistic regression showed: Among breast cancer patients of 40-49 years old group, HBcAb was positive in 648 cases (66.5%) and HBeAb positive in 344 cases (35.3%); Among controls in 40-49 years old group, HBcAb was positive in 372 cases (61.3%) and HBeAb positive in 167 patients (27.5%). The positive rates of HBcAb and HBeAb were significantly higher in breast cancer patients than those in controls (*p*<0.05) (Table 3).

**Table 3:** Comparison of HBV markers’ seropositivity in breast cancer patients and benign breast disease patients by age stratification

HBV markers		HBsAg		HBsAb		HBeAg		HBeAb		HBcAb	
		-	+	-	+	-	+	-	+	-	+
≤29 yrs	Benign breast disease patients	530	34	225	339	552	12	448	116	380	184
		(94.0%)	(6.0%)	(39.9%)	(60.1%)	(97.9%)	(2.1%)	(79.4%)	(20.6%)	(67.4%)	(32.6%)
	Breast cancer patients	32	4	15	21	36	0	23	13	18	18
		≠ (88.9%)	(11.1%)	(41.7%)	(58.3%)	(100%)	0.0%)	(63.9%)	(36.1%)	(50.0%)	(50.0%)
30-39 yrs	Benign breast disease patients	509	50	209	350	553	6	365	194	212	347
		(91.1%)	(8.9%)	(37.4%)	(62.6%)	(98.9%)	(1.1%)	(65.3%)	(34.7%)	(37.9%)	(62.1%)
	Breast cancer patients	295	26	139	182	314	7	215	106	132	189
		(91.9%)	(8.1%)	(43.3%)	(56.7%)	(97.8%)	(2.2%)	(67.0%)	(33.0%)	(41.1%)	(58.9%)
40-49 yrs	Benign breast disease patients	557	50	258	349	604	3	440	167*	235	372*
		(91.8%)	(8.2%)	(42.5%)	(57.5%)	(99.5%)	(0.5%)	(72.5%)	(27.5%)	(38.7%)	(61.3%)
	Breast cancer patients	884	90	403	571	968	6	630	344*	326	648*
		(90.8%)	(9.2%)	(41.4%)	(58.6%)	(99.4%)	(0.6%)	(65.0%)	(35.3%)	(33.7%)	(66.5%)
50-59 yrs	Benign breast disease patients	133	12	57	88	145	0	103	42	52	93
		(91.7%)	(8.3%)	(39.3%)	(60.7%)	(100.%)	(0%)	(71.%)	(29.%)	(35.9%)	(64.1%)
	Breast cancer patients	576	49	259	366	621	4	428	197	205	420
		(92.2%)	(7.8%)	(41.4%)	(58.6%)	(99.4%)	(0.6%)	(69.%)	(31.%)	(32.8%)	(67.2%)
≥60 yrs	Benign breast disease patients	47	4	21	30	51	0	40	11	12	39
		(92.2%)	(7.8%)	(41.2%)	(58.8%)	(100.%)	0%)	(78.4%)	(21.6%)	(23.5%)	(76.5%)
	Breast cancer patients	464	32	233	263	495	1	363	133	142	354
		(93.5%)	(6.5%)	(46.4%)	(53.6%)	(99.8%)	(0.2%)	(73.2%)	(26.8%)	(28.2%)	(71.8%)

* *p*<0.05

The average albumin concentration among breast cancer patients in the age groups of ≤29 years, 30-39 years, 40-49 years, 50-59 years and ≥60 years were 42.4 g/L, 41.7g/L, 41.6 g/L, 41.5 g/L, 40.9 g/L, respectively, which were significantly lower than those in controls (*p*<0.05); The mean ALT among breast cancer patients in the age groups of ≤29 years, 30-39 years, 40-49 years and 50-59 years were 21.5 U/L, 20.5 U/L, 22.4 U/L, 24.5 U/L, respectively, which were significantly higher than those in controls (*p*<0.05); Among breast cancer patients with 40-49 years age groups, the mean AST were 21.2 U/L, which were significantly higher than those in controls (*p*<0.05) (Table 4).

**Table 4:** Comparison of liver function in breast cancer patients and benign breast disease patients

Age	Groups	Albumin (g/L)	ALT(U/L)	AST(U/L)
≤29 yrs	Benign breast disease patients	45.4*	15.5*	18.5
	Breast cancer patients	42.4*	21.5*	18.7
30-39 yrs	Benign breast disease patients	43.8*	16.8*	18.6
	Breast cancer patients	41.7*	20.5*	19.6
40-49 yrs	Benign breast disease patients	43.3*	17.2*	19.3*
	Breast cancer patients	41.6*	22.4*	21.2*
50-59 yrs	Benign breast disease patients	43.6*	19.9*	21.8
	Breast cancer patients	41.5*	24.5*	23.2
≥60 yrs	Benign breast disease patients	41.5*	20.5	21.6
	Breast cancer patients	40.9*	23.6	23.9
Total	Benign breast disease patients	44.1*	16.8*	19.2*
	Breast cancer patients	41.4*	22.9*	22.0*

* *p*< 0.05

Of 1926 cases of controls, there were 67 cases of below 18 years old age, who had actively taken HBV vaccination in their neonatal period since the active HBV vaccination drive which was carried out in China in 1992. Among these patients, HBsAg was positive in 3 cases (4.5%), HBeAg positive in 3 cases (4.5%), HBcAb was positive in 10 cases (14.9%), HBsAb positive in 43 cases (64.2%), simple HBsAb positive in 34 cases (50.7%), HBeAb positive in 6 cases (9.0%) and all HBV markers were negative in 22 cases (32.8%) (Table 5).

**Table 5:** The distribution pattern of HBV infection in benign breast disease patients of ≤18 years old who had undergone HBV vaccination during their neonatal period

Serological markers of HBV					Benign breast disease patients (n=67)	
HBsAg	HBsAb	HBeAg	HBeAb	HBcAb	n	%
+	+/−	+/−	+/−	+/−	3	4.5%
+/−	+	+/−	+/−	+/−	43	64.2%
+/−	+/−	+	+/−	+/−	3	4.5%
+/−	+/−	+/−	+	+/−	6	9.0%
+/−	+/−	+/−	+/−	+	10	14.9%
-	+	-	-	-	34	50.7%
-	+	-	-	+	2	3.0%
-	+	-	+	+	5	7.5%
-	-	-	+	+	0	0
-	-	-	-	+	0	0
-	-	-	-	-	22	32.8%
+	-	-	+	+	0	0
+	-	+	-	+	2	3.0%
+	-	-	-	+	0	0
+	+	-	+	+	0	0
-	+	-	+	-	1	1.5%
+	+	+	-	+	1	1.5%

## DISCUSSION

About 15% of human cancers can be attributed to virus infection, and viruses are second only to tobacco as a risk factor for cancer [9]. Certain viruses are suspected to play a role in the initiation or promotion of breast cancer, including HCV [4–6, 10, 11]. HCV infection was reported to be associated with early-onset breast cancer [7], while HBV infection is more common than HCV in China, where every surgical patient should undergo routine examination of HBV serological markers and liver function tests for peri-operative preparation before operation. But current studies on the relationship between HBV infection and the risk of breast cancer are scanty at the moment.

In a case-control study to evaluate whether breast cancer in women is associated with chronic viral hepatitis infection conducted by Su et al [7], 1,958 patients with newly diagnosed breast cancer from the year 2000-2008 years and 7,832 subjects without cancer for comparison were included to identified HBV infection on the basis of the presence of HBsAg, and there was no significant difference in the prevalence of HBV infection between the breast cancer group (6.3%) and control group (6.1%). Notably, HBV relies on a retroviral replication strategy. For HBV-infected patients, eradication of HBV infection is rendered difficult because the stable covalently closed circular DNA (cccDNA) becomes established in hepatocyte nuclei and HBV DNA becomes integrated into the host genome indicating the presence of ongoing viral replication [12, 13]. After decades of years of acute HBV infection, even with the clearance of serum HBsAg and the presence of HBsAb, HBV DNA was still detected in liver, which means HBV replication may continue and persist for decades of years as an occult infection [14–16]. The majority of healthy individuals positive for HBcAb, which had been assumed to denote a past history of transient HBV infection, and HBV DNA was frequently detected in this crowd with negative for HBsAg but positive for HBcAb [17]. So screening of HBV infection by HBsAg may well was limited with the light on the potential impact that the HBV infection may have on the development of breast cancer. HBcAb may more accurately reflect a long-term subtle influence of HBV infection on the initiation or promotion of breast cancer. By contrast, our study registered 5 serological markers of HBV, including HBsAg and HBcAb. The positive rate of HBsAg in breast cancer patients was 8.2%, which was relatively higher than that (7.8%) in controls, but without significant difference (*P*>0.05). While, the positive rate of HBcAb (66.4%) in breast cancer patients was significantly higher than that (53.7%) in control group (*P*<0.05), so were the similar results in age groups of 40-49 years (*P*<0.05). It was indicated that previous infection or occult infection of HBV was more common in breast cancer patients, suggesting that exposure to HBV infection may be a risk factor for breast cancer and may be also related to the earlier age onset of breast cancer (peaked around 40-49 years) among Chinese females.

**Figure 2:**
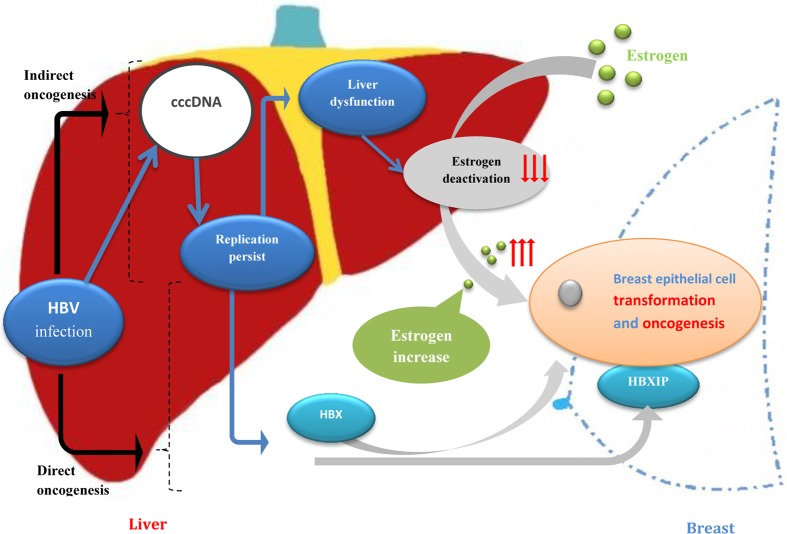
Potential mechanism of oncogenesis of breast epithelial cell caused by HBV infection

Cancer induction by HBV infection requires long periods of viral persistence usually covering for several decades [18]. Hepatocellular carcinoma (HCC) is well-known in the human neoplasms for being etiologically linked to HBV [19–21]. HBV infection can result in HBV replication without substantial liver injury for decades, but the risk of progression to cirrhosis and hepatocellular carcinoma maintained persistently over time [13]. Chronic HBV infection worldwide accounts for approximately 50% of all cases of hepatocellular carcinoma and virtually all childhood cases [19]. HBV X protein (HBX) interferes with cellular repair mechanisms may contribute to carcinogenesis [18]. Although the reasons of breast cancer promotion by HBV remain obscure and lack high-quality of epidemiological data, many researches *in vitro* have studied the function of HBX and hepatitis B X-interacting protein (HBXIP) on breast cancer cells [22–30]. Emerging evidence demonstrate that the HBX has been shown capable of transactivating many different viral and cellular promoters and high HBXIP expression was predominantly observed in breast cancer tissues instead of the adjacent normal breast tissues and HBXIP promotes the proliferation and migration of breast cancer cells and plays crucial roles in the development of breast cancer, serving as a key oncoprotein in cancer [22–30]. Furthermore, we presume a potential mechanism of indirect oncogenesis of breast epithelial cell caused by HBV infection: After HBV infection, HBV may persist as an occult infection and HBV replication may continue to cause a long-term subtle liver dysfunction [14–16]. Estrogen is mainly deactivated in liver and therefore estrogen may increase due to the result of liver dysfunction, while estrogen is a dominant risk factor of breast cancer, so final increase of gonadal hormone may promote the occurrence and development of breast cancer (Figure 2). In our result, the status of liver function in initially diagnosed breast cancer patients was significantly poorer than those in control group. Especially, all of albumin, ALT and AST were significantly poorer in 40-49 years old group, which was in accordance with HBcAb was significantly higher in 40-49 years old group. It suggest that the liver function of breast cancer patients was obviously poorer than controls at the time of initial diagnosis, which was in accordance with that potential mechanism of indirect oncogenesis of HBV infection on breast cancer. Certainly, various tumor related factors may also be related with liver dysfunction of breast cancer. This hypothesis was enlightened in our previous study about incidence of male breast cancer in Southwest of China, with a result that male breast cancer accounted for 1.96% to 6.5% (with the mean value of 2.9%) of breast cancers in Southwest of China from 2007 to 2011 and revealed that male breast cancer in Southwest of China was higher than that in United States(1%) [31]. So if oncogenesis of HBV in breast cancer functions through the impact on estrogen, and estrogen was also an important risk factor in the etiology of male breast cancer [32], males with an innate low-level of hormone may be more prone of having breast carcinoma through HBV infection than women. In addition, high HBV endemicity was more pronounced among males in china [33], so it is proved by our previous study that the rate of male breast cancer among all the breast cancers was relatively higher in Southwest of China [31].

Meanwhile, our results show that HBsAg positive group had not stronger association with breast cancer than those with HBcAb. There were some possible explanations. First, cancer induction by HBV infection requires long periods of viral persistence usually covering for several decades, HBsAg were not stable, permanent like HBcAb. So HBsAg positive patients not always have a stronger association with breast cancer than those with HBcAb. Second, the potential mechanism of oncogenesis of breast cancer caused by HBV infection was unclear. Breast cancer may cause by HBV infection from direct oncogenesis, and HBV may ensconce in breast tissue without expression of HBsAg. Third, this study sample size was not larger enough to get statistic difference of HBsAg may be also the reason.

The nationwide vaccination program since 1992 has changed the epidemiology of HBV infection in China from being high then to moderate endemic now. Perinatal infection with HBV may occur despite of the immunoprophylaxis, and mutations of HBV which leads to loss of immune epitopes and functional sites may be one of the important mechanisms [34]. A serological and molecular survey of HBV in children 15 years after inception of the national hepatitis B vaccination program in Eastern China indicated that the total rate of “a”-determinant mutations in the age 0–8 population was 13.72% at 15 years after the HBV vaccine was initially administered [35]. It has been reported that the prevalence of occult HBV infection is 10.9% in HBV vaccinated children in Taiwan [36]. In our study, among the controls of less than 18 years old who have undergone HBV vaccination at their neonatal period, HBsAg was positive in 3 cases (4.5%), HBcAb was positive in 10 cases (14.9%), and all of the HBV markers negative in 22 cases (32.8%). This data indicated that there had been still a high proportion of perinatal prophylactic failure despite the nationwide vaccination program drive carried out since 1992 in China. So the HBV screening and HBV vaccination efficacy monitoring should be enhanced from the neonatal period, which can probably prevent or delay the occurrence of breast cancer.

Strengths and limitations of our study should be considered. Strengths included the large size of the cohort, which was representative by a large segment of the Southwest China population, and complete record of serological markers of HBV and liver function tests. HBcAb may be more accurate to reflect a long-term subtle influence of HBV infection on the initiation or promotion of breast cancer than HBsAg. Indeed, for a sizeable subset of participants, we were able to analyze on the basis of age stratification. Unfortunately, our approach has several limitations. First, Family financial situation and History of intravenous drug abuse, what could affect transmission of HBV that mainly acquired through contaminated blood, may be potential confounding variable. We were regretful that the data of more potential confounding variable are incomplete. Second, we could not include additional serum markers (HBV DNA) that would show severity of HBV infection, and an additional healthy people group as control may improve the preciseness and creativeness of the results. HCV or HIV could be the potential confounding variables. If there is a disturbance, it's weak at best, because these infections are rare in China [37, 38]. Third, there is no neoteric national survey of HBV sero-epidemiology or available data of the prevalence of chronic HBV of our region. It will be helpful to give the readers a broader view of significance of HBV infection in your country.

In conclusion, current data about the relationship between HBV infection and the risk of breast cancer are scanty. Our study showed that the HBcAb seropositivity of breast cancer patients was significantly higher than that in the controls, so do the analysis on the basis of age stratification. So exposure to HBV infection may be a risk factor for breast cancer and may be related to earlier peak age of onset in breast cancer among Chinese females, and it is necessary to follow-up the immune responses to the hepatitis B vaccine. There has still been a high proportion of perinatal prophylactic failure despite the nationwide vaccination program drive carried out since 1992 in China. So the HBV screening and HBV vaccination efficacy monitoring should be enhanced from the neonatal period, which may probably prevent or delay the occurrence of breast cancer.

## MATERIALS AND METHODS

### Study population

This study was conducted in the Breast Cancer Center of Chongqing, the First Affiliated Hospital of Chongqing Medical University. There are approximately 31.4 million people who live in around 82402.95 km2 area of Chongqing, the Southwest of China. The medical records of 2471 cases of initially diagnosed breast cancer and 1951 cases of benign breast disease (as controls) were collected from January 2011 to march 2015. All of them had undergone surgical operation with pathological diagnosis. In China all the surgical patients should undergo routine examination of HBV serological markers and liver function tests for peri-operative preparation before operation. All of patients have signed an informed consent about allow their cases records be used for medical research and this study was approved by The Ethics Committee of the First Affiliated Hospital of Chongqing Medical University. Then, the clinical data of 2452 cases of initially diagnosed breast cancer and 1926 cases of controls with the consecutive reports of HBV serological markers and liver function tests, which were available in the Electronic Medical Records of the Breast Cancer Center of Chongqing, the First Affiliated Hospital of Chongqing Medical University, were enrolled in this study for comparative analysis. Benign breast diseases in the control group comprised of breast fibroadenoma, mastopathy, cyst of breast, intraductal papilloma of breast, and etc.

### Data collection

Blood samples were measured at The Clinical Laboratories of The First Affiliated Hospital of Chongqing Medical University(achieving ISO 15189 accreditation and College of American Pathologists Proficiency Test accreditation). The laboratory successfully completed the standardization and certification program. All the patients’ records including HBV serological markers and liver function tests were available in the Electronic Medical Records of the Breast Cancer Center of Chongqing, the First Affiliated Hospital of Chongqing Medical University. HBV serological makers include: HBsAg, Hepatitis B surface antibody (HBsAb), Hepatitis B e antigen (HBeAg), Hepatitis B e antibody (HBeAb), Hepatitis B core antibody (HBcAb). Liver function tests include: albumin, alanine aminotransferase (ALT), aspartate transaminase (AST).

### Statistical analysis

Significance in the differences of mean values across the age factor was assessed by Student's t test. The difference in the HBV markers’ seropositivity of all ages between breast cancer patients and benign breast disease patients were evaluated by Chi-square test. Data of HBV markers are number of cases and percentages. Because there were substantial differences in age among the two groups, to improve the precision, we conduct multiple layer analysis stratified by age and compare HBV markers after adjusted for age with binary logistic regression. The difference in the albumin, ALT, AST between breast cancer patients and benign breast disease patients in the same age groups were evaluated after adjusted for interactive of liver function and age with multivariate analysis of variance. Data of albumin, ALT, AST are mean ± SD. *P*<0.05 was considered statistically significant. All calculations were performed using the Statistical Package for Social Sciences software, version 15.0 (SPSS, Chicago, IL, USA).
